# Effect of Maternal Antidepressant Use During the Pre-pregnancy/Early Pregnancy Period on Congenital Heart Disease: A Prospective Cohort Study in Central China

**DOI:** 10.3389/fcvm.2022.916882

**Published:** 2022-07-05

**Authors:** Mengting Sun, Senmao Zhang, Yihuan Li, Letao Chen, Jingyi Diao, Jinqi Li, Jianhui Wei, Xinli Song, Yiping Liu, Jing Shu, Tingting Wang, Peng Huang, Jiabi Qin

**Affiliations:** ^1^Department of Epidemiology and Health Statistics, Xiangya School of Public Health, Central South University, Changsha, China; ^2^NHC Key Laboratory of Birth Defect for Research and Prevention, Hunan Provincial Maternal and Child Health Care Hospital, Changsha, China; ^3^Department of Cardiothoracic Surgery, Hunan Children's Hospital, Changsha, China; ^4^Guangdong Cardiovascular Institute, Guangdong Provincial People's Hospital, Guangdong Academy of Medical Sciences, Guangzhou, China; ^5^Hunan Provincial Key Laboratory of Clinical Epidemiology, Changsha, China

**Keywords:** antidepressant, pregnancy, offspring, congenital heart disease, risk factor

## Abstract

**Background:**

With the increase in maternal antidepressant prescribing before/during pregnancy, concerns about the safety of antidepressants have come into focus. The purpose of this study was to explore the association between maternal antidepressant use before pregnancy/in early pregnancy and the risk of congenital heart disease (CHD) in children, and to provide a scientific basis for clinical safety of antidepressant use.

**Methods:**

The prospective cohort study ultimately included 34,104 singleton pregnancies. Modified Poisson regression model with robust error variances was used to evaluate RRs and 95% confidence intervals (CIs) for the risk of CHD in offspring exposed to maternal antidepressant in the 3 months before pregnancy and early pregnancy. In addition, sensitivity analysis was further performed to explore the robustness of the results.

**Results:**

In this study, the maternal antidepressant exposure rate was 2.83% in the 3 months before pregnancy, 2.42% in early pregnancy, and the incidence of CHD was 8.973 per 1,000 live births. We found that maternal antidepressant use in the 3 months before pregnancy and early pregnancy were all associated with an increased risk of CHD, ~2.54 times and 2.87 times, respectively, of non-use of antidepressants after adjusting for potential confounders. This association was also found in CHD specific phenotypic analysis. Of these, offspring whose mothers were exposed to antidepressants in the 3 months before pregnancy had the highest risk of transposition of the great arteries (aOR = 5.50, 95% CI: 1.91–15.88). The offspring of mothers exposed to antidepressants in early pregnancy had the highest risk of developing ventricular septal defect (aOR = 4.80, 95% CI: 2.50–9.24). Sensitivity analysis verified the stability of the results.

**Conclusions:**

Maternal antidepressant use in the 3 months before pregnancy and early pregnancy were all associated with an increased risk of CHD in their offspring. In order to reduce the risk of teratogenesis, we recommend that pregnant women prepare for pregnancy after their condition improves or receive the minimum effective dose of medication.

## Introduction

Compared with men, women are more vulnerable to depression due to their differences in physiological anatomy, endocrine metabolism, social role and inner quality (1). This is especially true when a woman's pregnancy is accompanied by potential physical stress, putting her at an increased risk of depression. A recent meta-analysis reported that the prevalence of perinatal depression in China has shown a significant upward trend in recent 10 years, reaching 16.3% (95% CI: 14.7–18.2%), among which 19.7% (95%CI: 15.8–24.2%) was prenatal depression (2). As the number of pregnant women suffering from depression has increased significantly, so has the prescription of antidepressants. However, a new challenge arises: can antidepressants affect a developing fetus? Studies have reported the teratogenicity of antidepressants in early pregnancy, which have raised serious concerns about the safety of antidepressants used by mothers in early pregnancy, especially the risk of congenital heart defects in offspring (3, 4).

At present, congenital heart disease (CHD) is the most common birth defect in the world. It was reported that the prevalence of CHD was as high as 9.410 per 1,000 live births from 2010 to 2017, and is rising globally (5). The prevalence of CHD in China is 8.89 per 1,000 live births, close to the global incidence of CHD (6). In the long-term exploration of the pathogenesis and development of CHD, CHD is considered to be a lifelong disease (7), and children with CHD may be at high risk of long-term health risks such as brain damage, neurodevelopmental disorders, growth and development disorders or disability (8, 9). It is suggested that neonatal CHD seriously threatens the healthy development of mothers and infants, and is an important public health problem to be solved urgently in China. The etiological mechanism of CHD is complex. The current consensus is that CHD is the result of genetic and environmental factors as well as the interaction between the two, which may alter the structure of the heart by affecting the placenta (10). With the passionate study efforts of researchers, up to 400 genes associated with the pathogenesis of CHD have been found (11). However, the study reported that routine whole genome sequencing of a large number of CHD patients and their families can only provide a genetic explanation for 30% of CHD cases (12). In addition, considering that genetic factors are difficult to prevent and change, it is very important to find and identify environmental risk factors related to offspring CHD for the primary prevention of CHD.

Researchers have raised questions about the teratogenicity of antidepressant use during pregnancy after an epidemiological study conducted in 2005 found that paroxetine use during pregnancy increased the risk of heart malformations (13). Subsequent studies continue to explore the truth of this claim (14–16). A study has found that the use of fluoxetine in early pregnancy increases the risk of abnormal pulmonary venous reflux in offspring [OR = 2.56; 95% confidence interval (CI): 1.10–5.93] (17). A large meta-analysis of more than 6 million people found that sertraline use in early pregnancy significantly increased the risk of CHD in offspring (OR = 1.36, 95% CI: 1.06–1.74) (18). A recent meta-analysis also showed an odds ratio of 1.28 (95% CI: 1.17–1.41) between use of any antidepressant in the first trimester of pregnancy and congenital heart defects in the offspring (19). However, this association may be due to confusion about maternal characteristics, such as lifestyle, diet, personal history of illness, and exposure to other risk factors, resulting in inaccurate risk estimates.

Prohibition of antidepressants during pregnancy is of course an ideal state, but it may lead to the aggravation of depression related diseases and even suicidal tendencies (20). Therefore, it is particularly important to elucidate the relationship between antidepressant use and CHD risk in pregnant women. We believe that different races may have different results due to great differences in various aspects. Considering the impact of pharmacokinetics, there is no evidence of the association between maternal antidepressant use before pregnancy and the risk of CHD in offspring. Here, we constructed a prospective cohort study to evaluate the association between antidepressant use (in 3 months before pregnancy and in early pregnancy, respectively) and total CHD and its specific phenotype in offspring for the first time in China.

## Materials and Methods

### Ethics Statement

This study is a prospective cohort study on maternal and child health in Hunan province based on Hunan Maternal and Child Health Hospital. This study was approved by the Ethics Committee of Xiangya School of Public Health, Central South University and registered in The Chinese Clinical Trial Registry (Registration number: ChiCTR1800016635). All subjects were informed of the study protocol in detail and provided written informed consent before entering the cohort, and the ethical principles of the Declaration of Helsinki of the World Medical Association were strictly followed in this study.

### Study Population and Information Collection

The cohort was conducted in Hunan Maternal and Child Health Hospital from March 13, 2013 to December 31, 2019. Hunan Maternal and Child Health Hospital is a class A, grade III hospital with a long history, located in Hunan Province, central China. All subjects were enrolled in the cohort at the time of their first prenatal care. For study purposes, we excluded pregnant women using assisted reproductive technology, pregnant women with multiple pregnancies, and offspring diagnosed with chromosomal abnormalities or syndromic CHD. Finally, a total of 34,104 pregnant women were included in the analysis.

In this study, we designed a structured questionnaire, including the sociodemographic characteristics of the subjects, obstetric and reproductive history, lifestyle, other health-related factors and health information of their offspring. In order to ensure the accuracy of the collected research data, our investigators underwent strict unified training in accordance with the standard way before the field investigation, and then completed the questionnaire survey through face-to-face interview with our participants. The relevant health information of the offspring was completed by telephone follow-up 3 months after delivery and reconfirmed in the medical record system of the hospital.

### Exposures

To assess exposure, information on the prescribing of antidepressants by mothers in the 3 months before pregnancy and early pregnancy was collected. The prescribing information was obtained from the pregnant women's prescriptions and secondary confirmations from the hospital's medical system. The information was collected by the investigator when conducting face-to-face interviews. First, the investigator asked the pregnant women whether they had received antidepressant drugs. If so, the pregnant women were required to provide a doctor's prescription or confirm by querying the hospital's electronic medical records system. If the medicine was not prescribed in this hospital, let pregnant women query the medical records by logging in to the hospital's app or Wechat applet. Women who used only prescribed 1 consistent antidepressant in the 3 months before pregnancy and early pregnancy were considered to be the exposure group. Antidepressant use was defined as at least one prescription for an oral antidepressant drug during the exposure period, which ranged from the 3 months before pregnancy to the beginning of pregnancy, or the day of pregnancy initiation up to the end of the first 3 months of gestation. This avoided the confusion of subsequent results caused by the simultaneous use of multiple antidepressants and the replacement of antidepressants.

### Diagnosis of CHD

In this study, CHD was defined as malformation of heart cavities, heart valves, great arteries, veins, and septal defects (ICD10: Q20.0–Q28.0). Infants with CHD were diagnosed by neonatal echocardiography. Seven CHD phenotypes were evaluated, including atrial septal defect (ASD), ventricular septal defect (VSD), atrioventricular septal defect (AVSD), patent ductus arteriosus (PDA), Tetralogy of Fallot (TOF), pulmonary stenosis (PS), and transposition of the great arteries (TGA) (21). The ICD diagnostic codes corresponding to each CHD phenotype were shown in Table 1.

**Table 1:** Prevalence of CHD in offspring.

**Type of CHD**	**ICD-10 diagnosis codes**	**Number of cases**	**Prevalence (95% CI)**
Total CHD	–	306	8.97‰ (7.97–9.97‰)
ASD	Q21.0	86	2.52‰ (1.99–3.05‰)
VSD	Q21.1	105	3.08‰ (2.49–3.67‰)
AVSD	Q21.2	6	0.18‰ (0.04–0.32‰)
PDA	Q25.0	50	1.47‰ (1.06–1.87‰)
TOF	Q21.3	24	0.70‰ (0.42–0.99‰)
PS	Q25.6	36	1.06‰ (0.71–1.40‰)
TGA	Q20.3	28	0.82‰ (0.52–1.13‰)

*ASD, atrial septal defect; AVSD, atrioventricular septal defect; CHD, congenital heart disease; PDA, patent ductus arteriosus; PS, pulmonary stenosis; TGA, transposition of the great arteries; TOF, tetralogy of fallot; VSD, ventricular septal defect*.

### Covariates

In this study, the information about covariates involved maternal sociodemographic characteristics, obstetric and reproductive history, lifestyle before and during pregnancy, and other health-related factors. Among them, sociodemographic characteristics include maternal age, education level, main residence, and nationality. The maternal age was divided into <35 years old and ≥35 years old (advanced maternal age). The education level was divided into four categories, including junior high and below, high school or technical secondary school, junior college and Bachelor degree or above. The main areas of residence were urban and rural areas. Ethnic groups were divided into Han and minorities (55 ethnic minorities except Han). Obstetric and reproductive history included consanguineous marriage (i.e., yes or no), family history of congenital malformation (i.e., yes or no), history of gestational diabetes mellitus (GDM) (i.e., yes or no) and history of gestational hypertension (GH) (i.e., yes or no). Lifestyle includes pre-pregnancy/early pregnancy smoking (i.e., yes or no) and pre-pregnancy/early pregnancy alcohol consumption (i.e., yes or no). Alcohol consumption was defined as consuming at least one standard alcoholic beverage during exposure, and smoking was defined as smoking at least one cigarette during exposure. Health related factors included maternal pre-pregnancy body mass index (BMI), folic acid use in 3 months before pregnancy or in early pregnancy (i.e., yes or no), house decoration in 3 months before pregnancy or in early pregnancy (i.e., yes or no), and exposure to radioactive substances at work in 3 months before pregnancy or in early pregnancy (i.e., yes or no). Pre-pregnancy BMI was divided into four categories according to the appropriate BMI for Asians recommended by the World Health Organization, including <18.5 kg/m^2^ (underweight), 18.5–<23.0 kg/m^2^ (normal weight), 23.0– <27.5 kg/m^2^ (overweight) and ≥27.5 kg/m^2^ (obesity).

### Statistical Analysis

**Figure 1:**
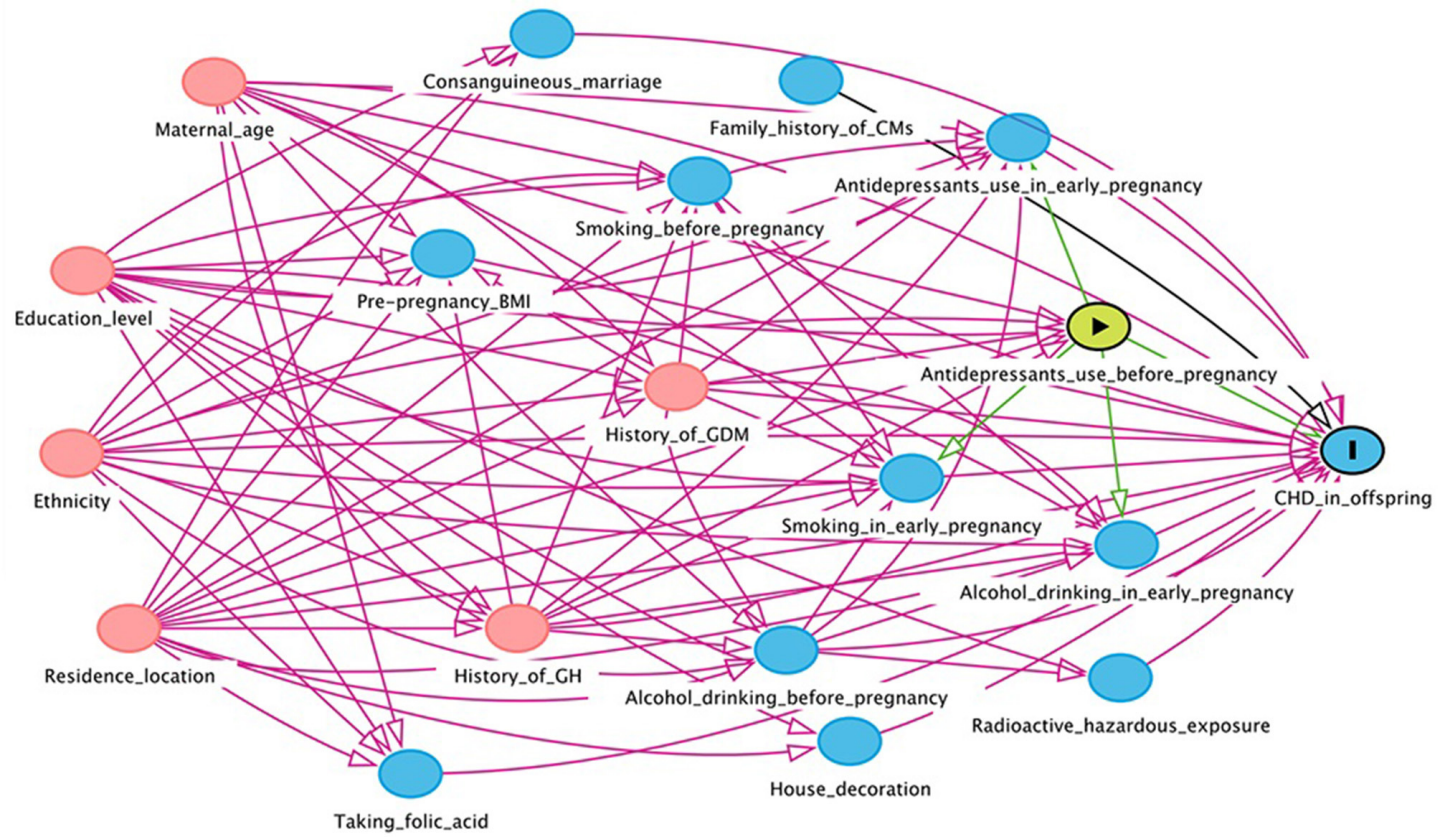
Directed acyclic graph for the association between maternal antidepressant use in 3 months before pregnancy and risk of CHD in offspring. Red arrows indicate biasing paths, green arrows indicate causal paths. Minimal sufficient adjustment sets for estimating the effect of maternal antidepressant use in 3 months before pregnancy on offspring CHD: maternal age, ethnicity, residence location, education level, history of gestational diabetes mellitus and history of gestational hypertension. BMI, body mass index; CHD, congenital heart disease; CM, congenital malformation; GDM, gestational diabetes mellitus; GH, gestational hypertension.

**Figure 2:**
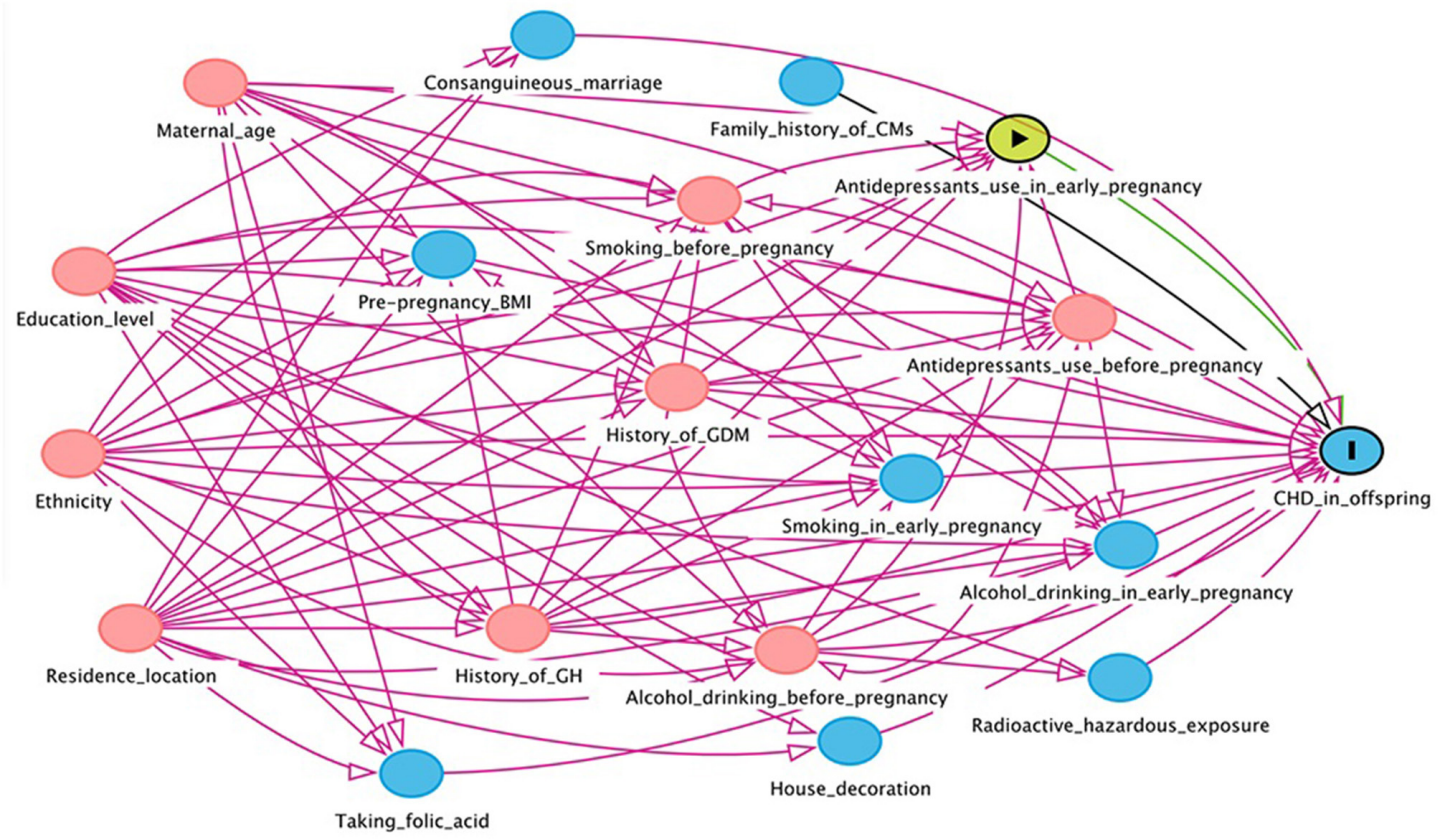
Directed acyclic graph for the association between maternal antidepressant use in early pregnancy and risk of CHD in offspring. Red arrows indicate biasing paths, green arrows indicate causal paths. Minimal sufficient adjustment sets for estimating the effect of maternal antidepressant use in early pregnancy on offspring CHD: maternal age, ethnicity, residence location, education level, history of gestational diabetes mellitus, history of gestational hypertension, smoking before pregnancy, alcohol drinking before pregnancy and antidepressants use before pregnancy. BMI, body mass index; CHD, congenital heart disease; CM, congenital malformation; GDM, gestational diabetes mellitus; GH, gestational hypertension.

In this study, EpiData version 3.1 was used to input the collected maternal and infant health data. In order to ensure the preciseness of the data, the double entry mode was adopted. The Directed Acyclic Graph (DAG) was used to construct a causal network based on the offspring CHD theory to identify variables that might confound the association between maternal antidepressant use and offspring CHD risk (Figures 1, 2). DAG was built through DAGitty online software (22).

We assessed sociodemographic distribution and maternal characteristics based on antidepressant use and CHD, respectively. Comparisons between categorical variables were tested using χ^2^ tests and Fisher's exact probability. Poisson regression model is a common model for influencing factors of rare disease occurrence frequency, which is mainly used to analyze prospective data such as cohort follow-up. When poisson regression is applied to binomial data, the error of estimation relative risk is overestimated. Zou proposed to rectify this by using a robust error variance procedure known as sandwich estimation, that is, Modified Poisson regression model (23). In this study, Modified Poisson regression model with robust error variances was used to evaluate RRs and 95% confidence intervals (CIs) for the risk of CHD in offspring exposed to maternal antidepressant in the 3 months before pregnancy and early pregnancy. In addition to the risk of total CHD in offspring, we further analyzed the phenotypes of CHD (i.e., ASD, VSD, AVSD, PDA, TOF, PS and TGA). Since the number of AVSD, TOF and PS events in the exposed group was zero, they were not taken into account. Finally, we analyzed the association between maternal antidepressant use in the 3 months before pregnancy/early pregnancy and total CHD and its four phenotypes (i.e., ASD, VSD, PDA and TGA) in the offspring. Among them, the crude RRs and their corresponding 95% CI were obtained without any adjustment in Model 1; minimum sufficient adjustment set obtained in DAG analysis was adjusted in Model 2; smoking and alcohol drinking in the 3 months before pregnancy (or smoking and alcohol drinking in early pregnancy and antidepressants use before pregnancy) were further adjusted in Model 3 on the basis of adjusting the minimum sufficient adjustment set.

We further performed two sensitivity analyses to explore the robustness of the results. The association between maternal antidepressant use and offspring CHD was reanalyzed by excluding women whose offspring had any congenital malformations other than CHD or women whose offspring had more than one CHD phenotype. Two-sided *P* < 0.05 was considered statistically significant. All analyses were completed in SPSS 26.0 software, and forest map was completed by R 4.1.2 software.

## Results

### Characteristics of the Study Objects

A total of 34,104 pregnant women were included in the final analysis of this study. Among them, we identified 964 (2.83%) mothers took antidepressant prescription in the 3 months before pregnancy and 824 (2.42%) mothers took antidepressant prescriptions in early pregnancy. After follow-up and secondary confirmation of medical records, 306 fetuses were diagnosed with non-syndromic CHD (the incidence of CHD was 8.973 per 1,000 live births), including 86 with ASD, 105 with VSD, 6 with AVSD, 50 with PDA, 24 with TOF, 36 with PS and 28 with TGA. It is worth noting that some cases have been diagnosed with multi-phenotypic CHD. Therefore, the sum of phenotypes was not equal to 306. The incidence of each type of CHD was shown in Table 1. The distribution of baseline characteristics according to the status of maternal antidepressant use in 3 months before pregnancy as well as in early pregnancy is summarized in Table 2.

**Table 2:** The distribution of baseline characteristics according to status of maternal antidepressant use before pregnancy or in early pregnancy.

**Variables**	**Total, No. (%)** **(*N* = 34,104)**	**Antidepressants use before pregnancy**		**Antidepressants use in early pregnancy**	
		**No** **(*n* = 33,140)**	**Yes** **(*n* = 964)**	**No** **(*n* = 33,280)**	**Yes** **(*n* = 824)**
**Maternal age**					
<35 years	26,445 (77.5)	25,715 (77.6)	730 (75.7)	25,810 (77.6)	635 (77.1)
≥35 years	7,659 (22.5)	7,425 (22.4)	234 (24.3)	7,470 (22.4)	189 (22.9)
**Maternal education level**					
Junior high and below	2,547 (7.5)	2,465 (7.4)	82 (8.5)	2,496 (7.5)	51 (6.2)
High school or technical secondary school	9,695 (28.4)	9,409 (28.4)	286 (29.7)	9,471 (28.5)	224 (27.2)
Junior college	15,594 (45.7)	15,151 (45.7)	443 (46.0)	15,218 (45.7)	376 (45.6)
Bachelor degree or above	6,268 (18.4)	6,115 (18.5)	153 (15.9)	6,095 (18.3)	173 (21.0)
**Residence location**					
Urban areas	21,074 (61.8)	20,460 (61.7)	614 (63.7)	20,552 (61.8)	522 (63.3)
Rural areas	13,030 (38.2)	12,680 (38.3)	350 (36.3)	12,728 (38.2)	302 (36.7)
**Ethnicity**					
Han	33,656 (98.7)	32,708 (98.7)	948 (98.3)	32,845 (98.7)	811 (98.4)
Minority	448 (1.3)	432 (1.3)	16 (1.7)	435 (1.3)	13 (1.6)
**Pre-pregnancy BMI categories**					
<18.5 kg/m^2^	4,920 (14.4)	4,765 (14.4)	155 (16.1)	4,796 (14.4)	124 (15.0)
18.5– <23.0 kg/m^2^	21,056 (61.7)	20,482 (61.8)	574 (59.5)	20,554 (61.8)	502 (60.9)
23.0– <27.5 kg/m^2^	6,963 (20.4)	6,757 (20.4)	206 (21.4)	6,792 (20.4)	171 (20.8)
≥27.5 kg/m^2^	1,165 (3.4)	1,136 (3.4)	29 (3.0)	1,138 (3.4)	27 (3.3)
**Smoking before pregnancy**					
No	33,762 (99.0)	32,818 (99.0)	944 (97.9)	32,942 (99.0)	820 (99.5)
Yes	342 (1.0)	322 (1.0)	20 (2.1)	338 (1.0)	4 (0.5)
**Alcohol drinking before pregnancy**					
No	33,524 (98.3)	32,586 (98.3)	938 (97.3)	32,710 (98.3)	814 (98.8)
Yes	580 (1.7)	554 (1.7)	26 (2.7)	570 (1.7)	10 (1.2)
**Smoking in early pregnancy**					
No	33,650 (98.7)	32,706 (98.7)	944 (97.9)	32,832 (98.7)	818 (99.3)
Yes	454 (1.3)	434 (1.3)	20 (2.1)	448 (1.3)	6 (0.7)
**Alcohol drinking in early pregnancy**					
No	33,606 (98.5)	32,666 (98.6)	940 (97.5)	32,794 (98.5)	812 (98.5)
Yes	498 (1.5)	474 (1.4)	24 (2.5)	486 (1.5)	12 (1.5)
**Consanguineous marriage**					
No	33,956 (99.6)	32,996 (99.6)	960 (99.6)	33,134 (99.6)	822 (99.8)
Yes	148 (0.4)	144 (0.4)	4 (0.4)	146 (0.4)	2 (0.2)
**History of gestational diabetes**					
No	31,280 (91.7)	30,398 (91.7)	882 (91.5)	30,504 (91.7)	776 (94.2)
Yes	2,824 (8.3)	2,742 (8.3)	82 (8.5)	2,776 (8.3)	48 (5.8)
**History of gestational hypertension**					
No	31,816 (93.3)	30,920 (93.3)	896 (92.9)	31,048 (93.3)	768 (93.2)
Yes	2,288 (6.7)	2,220 (6.7)	68 (7.1)	2,232 (6.7)	56 (6.8)
**Family history of congenital malformations**					
No	34,042 (99.8)	33,082 (99.8)	960 (99.6)	33,222 (99.8)	820 (99.5)
Yes	62 (0.2)	58 (0.2)	4 (0.4)	58 (0.2)	4 (0.5)
**Taking folic acid in 3 months before pregnancy or in early pregnancy**					
No	32,556 (95.5)	31,646 (95.5)	910 (94.4)	31,776 (95.5)	780 (94.7)
Yes	1,548 (4.5)	1,494 (4.5)	54 (5.6)	1,504 (4.5)	44 (5.3)
**House decoration in 3 months before pregnancy or in early pregnancy**					
No	32,510 (95.3)	31,584 (95.3)	926 (96.1)	31,722 (95.3)	788 (95.6)
Yes	1,594 (4.7)	1,556 (4.7)	38 (3.9)	1,558 (4.7)	36 (4.4)
**Exposure to radioactive hazardous while at work in 3 months before pregnancy or in early pregnancy**					
No	32,980 (96.7)	32,058 (96.7)	922 (95.6)	32,186 (96.7)	794 (96.4)
Yes	1,124 (3.3)	1,082 (3.3)	42 (4.4)	1,094 (3.3)	30 (3.6)

*BMI, body mass index*.

### Maternal Antidepressant Use in 3 Months Before Pregnancy and Risk of CHDs in Offspring

We constructed DAGs to identify potential causal frameworks and covariables for maternal antidepressant use in 3 months before pregnancy/antidepressant use in early pregnancy-CHDs associations. In the maternal antidepressant use in 3 months before pregnancy-CHDs associations, we considered 14 covariates and retained 6 based on our DAG (covariates selected by DAG are presented in the footnotes of Figure 1). In the maternal antidepressant use in early pregnancy-CHDs associations, we further considered the three covariates of alcohol drinking in early pregnancy, smoking in early pregnancy and maternal antidepressant use before pregnancy. In total, we considered 17 covariates and retained 9 based on our DAG (covariates selected by DAG are presented in the footnotes of Figure 2). We obtained a minimum sufficient adjustment set to estimate the effect of maternal antidepressant use in 3 months before pregnancy on offspring CHD including 6 covariates including maternal age, ethnicity, residence location, education level, history of gestational diabetes mellitus and history of gestational hypertension. In the minimum sufficient adjustment set to estimate the effect of maternal antidepressant use in early pregnancy on offspring CHD, 3 covariables including smoking before pregnancy, alcohol drinking before pregnancy and antidepressants use before pregnancy were added on the basis of the 3 months before pregnancy.

**Figure 3:**
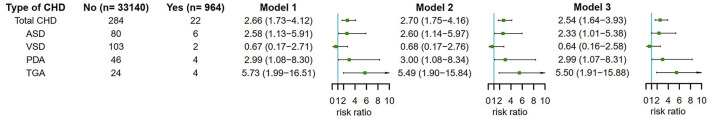
Maternal antidepressant use in 3 months before pregnancy and risk of CHD and its phenotypes in offspring. Model 1 was a crude model without any variable adjusted. Model 2 adjusted for maternal age, ethnicity, residence location, education level, history of gestational diabetes mellitus and history of gestational hypertension. Model 3 adjusted for maternal age, ethnicity, residence location, education level, history of gestational diabetes mellitus, history of gestational hypertension, smoking before pregnancy and alcohol drinking before pregnancy. CHD, congenital heart disease.

In the overall population, mothers who used antidepressant in 3 months before pregnancy had an increased risk of CHD in their offspring compared with mothers without used antidepressant. The adjusted RR of total CHDs was 2.54 (95% CI, 1.64–3.93) for maternal antidepressant use in 3 months before pregnancy after adjusting for maternal age, ethnicity, residence location, education level, history of GDM, history of GH, smoking before pregnancy and alcohol drinking before pregnancy. To understand whether maternal antidepressant use before pregnancy has different effects on offspring CHD phenotypes, we further analyzed 4 different CHD phenotypes. Similarly, we found that maternal antidepressant use in 3 months before pregnancy was independently associated with ASD, PDA, and TGA in offspring in model 3, and the corresponding adjusted RR was 2.33 (95% CI, 1.01–5.38) for ASD, 2.99 (95% CI, 1.07–8.31) for PDA and 5.50 (95% CI, 1.91–15.88) for TGA respectively (Figure 3).

### Maternal Antidepressant Use in Early Pregnancy and Risk of CHDs in Offspring

**Figure 4:**
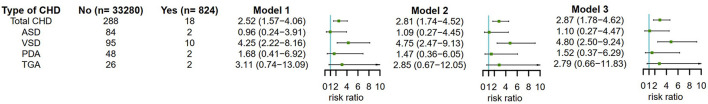
Maternal antidepressant use in early pregnancy and risk of CHD and its phenotypes in offspring. Model 1 was a crude model without any variable adjusted. Model 2 adjusted for maternal age, ethnicity, residence location, education level, history of gestational diabetes mellitus, history of gestational hypertension, smoking before pregnancy, alcohol drinking before pregnancy and antidepressants use before pregnancy. Model 3 adjusted for maternal age, ethnicity, residence location, education level, history of gestational diabetes mellitus, history of gestational hypertension, smoking before pregnancy, alcohol drinking before pregnancy, antidepressants use before pregnancy, smoking in early pregnancy and alcohol drinking in early pregnancy. CHD, congenital heart disease.

Then, we analyzed the association between maternal antidepressant use in early pregnancy and CHD risk in offspring (Figure 4). Encouragingly, we also found that maternal antidepressant use in early pregnancy significantly increased the risk of total CHDs in offspring. The adjusted RR of total CHDs was 2.87 (95% CI, 1.78–4.62) for maternal antidepressant use in early pregnancy after adjusting for maternal age, ethnicity, residence location, education level, history of GDM, history of GH, smoking before pregnancy, alcohol drinking before pregnancy, antidepressants use before pregnancy, smoking in early pregnancy and alcohol drinking in early pregnancy. In CHD phenotypic analysis, maternal antidepressant use in early pregnancy was only significantly associated with VSD in offspring in Model 3, and the corresponding adjusted RR was 4.80 (95% CI, 2.50–9.24). We speculated that it might be due to the small sample size.

### Sensitivity Analysis

**Figure 5:**
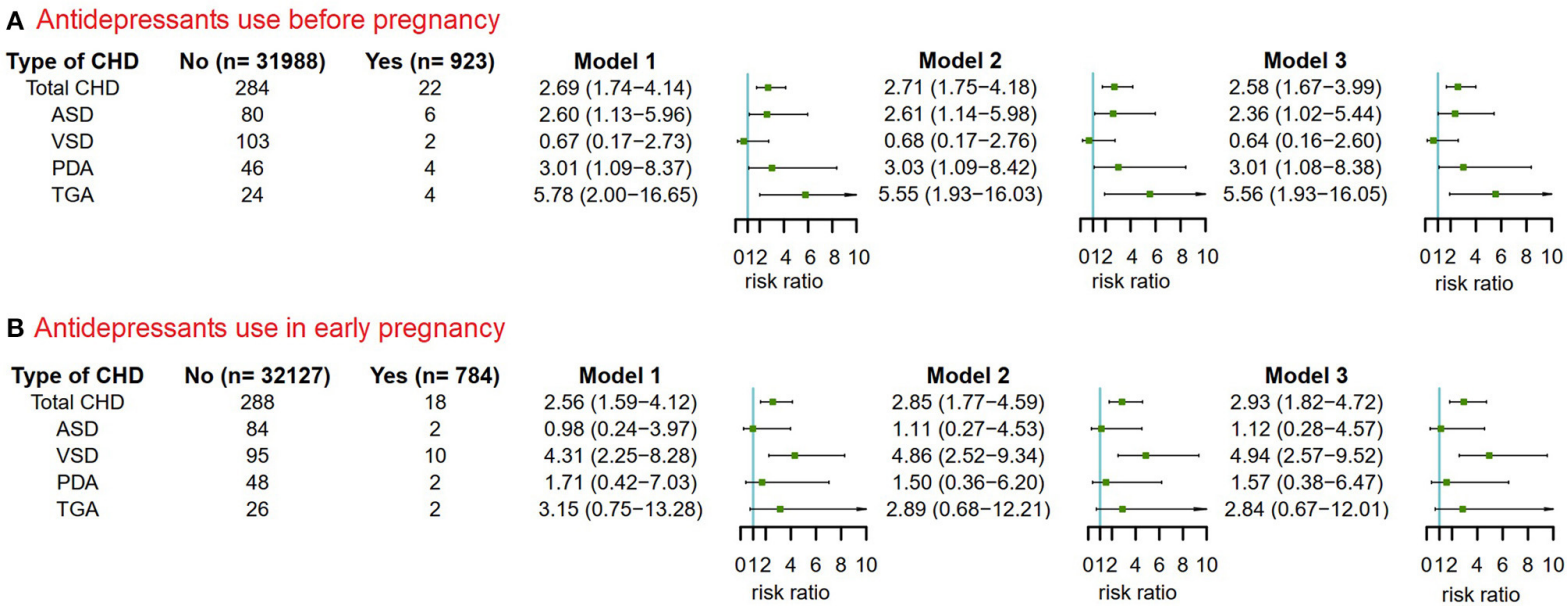
The risks of CHD in offspring of pregnant women exposured to antidepressant in 3 months before pregnancy and in early pregnancy after excluding pregnant women whose children had non-cardiac defects. Model 1 was a crude model without any variable adjusted. Model 2 adjusted for maternal age, ethnicity, residence location, education level, history of gestational diabetes mellitus and history of gestational hypertension. Model 3 adjusted for maternal age, ethnicity, residence location, education level, history of gestational diabetes mellitus, history of gestational hypertension, smoking before pregnancy and alcohol drinking before pregnancy. CHD, congenital heart disease. **(A)** Antidepressants use before pregnancy. **(B)** Antidepressants use in early pregnancy.

**Figure 6:**
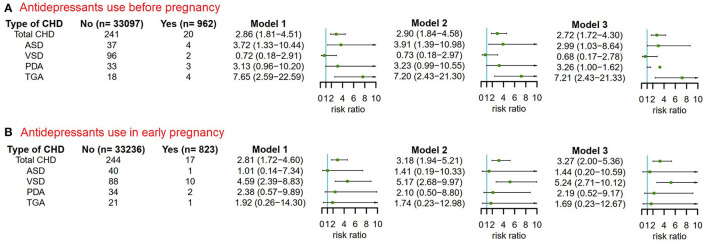
The risks of CHD in offspring of pregnant women exposured to antidepressant in 3 months before pregnancy and in early pregnancy after excluding pregnant women whose children were diagnosed with more than one CHD phenotypes. Model 1 was a crude model without any variable adjusted. Model 2 adjusted for maternal age, ethnicity, residence location, education level, history of gestational diabetes mellitus, history of gestational hypertension, smoking before pregnancy, alcohol drinking before pregnancy and antidepressants use before pregnancy. Model 3 adjusted for maternal age, ethnicity, residence location, education level, history of gestational diabetes mellitus, history of gestational hypertension, smoking before pregnancy, alcohol drinking before pregnancy, antidepressants use before pregnancy, smoking in early pregnancy and alcohol drinking in early pregnancy. CHD, congenital heart disease. **(A)** Antidepressants use before pregnancy. **(B)** Antidepressants use in early pregnancy.

The reliability of the previous results was shown in the results of two sensitivity analyses that excluded women whose children had any congenital malformations other than CHD (Figure 5) or whose children had more than one CHD phenotype (Figure 6).

## Discussion

Previous studies have suggested that antidepressant use by pregnant women may increase the risk of CHD in their offspring. As most of the studies were analyzed the data from developed countries, our study is the first to explore this association using data from Hunan province in central China. This prospective cohort study was conducted from March 13, 2013 to December 31, 2019 at Hunan Maternal and Child Health Hospital. In our study, the incidence of CHD was 8.97 per 1,000 live births (306/34,104). We found that maternal antidepressant use in 3 months before pregnancy and early pregnancy were associated with a significant increase in CHD in offspring, with adjusted RRs of 2.54 (95% CI, 1.64–3.93) and 2.87 (95% CI, 1.78–4.62), respectively. In CHD-specific phenotypic analysis, maternal antidepressant use in the 3 months before pregnancy significantly increased the risk of ASD, PDA, and TGA in children, with having a highest estimated risk of 5.50 for TGA. However, maternal antidepressant use in early pregnancy increased the risk for VSD by ~4.80 times that of the group not using antidepressant in early pregnancy. Sensitivity analysis verified the stability of the results. We believe that the difference between pre-pregnancy and early pregnancy phenotype results may be due to the fact that different phenotypes may have different etiologies and, conceivably, different risks for each phenotype. In addition, the number of cases for each CHD phenotype is very small and there may not be sufficient statistical detection capacity available.

In China, the prescription of antidepressants for pregnant women is conservative, and there are many pregnant women who take antidepressants before pregnancy and stop treatment after pregnancy. However, there were few studies on the adverse pregnancy outcomes of pre-pregnancy antidepressant use in children. This study was the first to provide strong evidence that maternal antidepressant use in the 3 months before pregnancy increased the risk of CHD in children. Therefore, we call for attention to avoid the risk factors related to the adverse pregnancy outcomes of the offspring as much as possible during the preparation of pregnancy, and try to choose pregnancy when the mother's personal disease improves, which is also one of the best choices for healthy birth and healthy education.

A recent meta-analysis based on 20 studies from 14 countries was consistent with our study, finding that mothers who used any antidepressant in early pregnancy were 1.2 to 1.4 times more likely to have a child with a congenital heart defect than those who did not (19). This meta-analysis limited the high quality of all studies through strict inclusion criteria, and the results had low heterogeneity. In addition, the largest current meta-analysis from Shen et al., involving more than 6 million pregnant women, found an increased risk of atrial and/or ventricular septal defects in infants using sertraline (18), which is similar to our analysis of CHD specific phenotypes. In addition, a study from the Danish Registry reported an increased risk of severe heart malformations from early pregnancy exposure to venlafaxine, but the absolute risk was low (24). One point of concern is that the risk may differ for each class of antidepressant, so more research is needed to explore the differences.

There were still some studies that put forward the opposite view. Kayla et al. found some association between maternal antidepressant use and CHD, but this association weakened after partially accounting for underlying conditions (17). In a cohort study of over 60,000 pregnant women, Krista et al. found that the association between antidepressants in early pregnancy and cardiac malformations decreased as the level of confounder adjustment increased, suggesting that antidepressant use in early pregnancy did not significantly increase the risk of cardiac malformations. However, antidepressant exposure rates in this cohort (6.80%) were much higher than in our study (2.42%) (25). Few Asian countries have studied this association, and a retrospective cohort study in Japan found no increased risk of total congenital malformations in children whose mothers used antidepressants of any type in early pregnancy (aOR = 0.86; 95% CI: 0.52–1.42). In addition, there was no increased risk of organ-specific abnormalities in the analysis by antidepressant type. However, total antidepressant use in the study was only 0.7% (392/53,638) (26). This may be related to the background that the teratogenic potential of drugs became widely recognized after the harmful effects of thalidomide occurred in Japan in the 1960s (27).

This study identified a significant association between maternal antidepressant use and increased CHD risk in children. However, the potential biological mechanisms involved in the association between maternal antidepressant use and offspring CHD remain to be elucidated. In recent years, ion channels as target receptors for antidepressant drugs have attracted much attention. One potential mechanism is the delayed-rectifier potassium current (I_K_), which is a common teratogenic mechanism. I_K_ includes I_Kr_, which activates quickly, and I_Ks_, which activates slowly (28). The K_r_ (KCNH2 gene) and K_s_ ion channel (KCNQ1 + KCNE1 gene) are highly expressed in human embryos and adult hearts (29). Genetic mutations in the human subunits that form the K_r_ and K_s_ channels prolong cardiac action potentials (AP), leading to a prolonged QT interval, known as long QT syndrome (30, 31). Recent epidemiological studies have reported an association between antidepressants with a risk of long QT syndrome and cardiac defects. The use of antidepressants cimipramil (32) and clomiphene (33) has been identified to double the risk of ventricular-septal defects. In animal experiments, it has also been found that several drugs that block the K_r_ channel can lead to malformations (including heart defects) and embryo death (29).

Currently, there is an alternative view on the biological mechanism between antidepressants with selective serotonin reuptake inhibitors (SSRIs) and CHD. Serotonin (5-HT) is a neurotransmitter that plays an important role in embryonic development as a signaling molecule (34). It is also reported that 5-HT may be involved in cardiac development, such as progenitor cell formation and outflow tract lengthening (35–37). SSRIs can cross the placental barrier and block 5-HT reuptake, which provides biological justification for their teratogenic effect on heart development (38). However, there is a trend of diversification of drugs on the market, with each drug exhibiting its own unique receptor binding profile (39). Therefore, there may be different complex mechanisms that need to be explored by a large number of animal experiments, cell experiments and epidemiological studies.

## Limitation

In this study, exposure information was collected prospectively to control for recall bias as much as possible. In addition, many confounding factors, including women's lifestyle and obstetric history, were adjusted. However, there were still several limitations in this study. The data collected in this study came from only one hospital, which inevitably resulted in the deviation of admission rate. The number of cases in this study for some CHD phenotypes was very small and may not have obtained sufficient statistical detection capabilities. However, considering that CHD is a rare disease with a low incidence, and the incidence of its subtypes is even lower, it is difficult to avoid a small number of cases in the cohort study of influencing factors of CHD. We will continue to collect samples to expand the number of cases. In addition, we analyzed the relationship between exposure and CHD by Modified Poisson regression, which can provide more stable estimates. In this study, we did not focus on the dose of medication to determine the effect of persistent or intermittent medication patterns on CHD. We only paid attention to frequency, which is the limitation of this study. Different antidepressants may have different teratogenic effects, and this study also failed to record the types of drugs prescribed. However, the number of women using antidepressants during pregnancy in this study was relatively small, and we may not be able to ensure a sufficient sample size to conduct a more detailed analysis in subgroups. Despite the abundance of information available to adjust for confounders, there is always the possibility of residual confounders in observational studies. Therefore, sensitivity analysis was used to minimize this possibility. The fetuses of the mothers included in this study were all live births, so it was inevitable that the parents may terminate pregnancy when they find that there may be adverse pregnancy outcomes, so some cases or events would be missed.

## Conclusion

In conclusion, it is of concern that antidepressant use in the 3 months before pregnancy and in the early pregnancy may significantly increase the risk of CHD in children. According to the results of our study, we suggest that women should avoid taking some teratogenic drugs as much as possible during pregnancy preparation, and try to prepare for pregnancy after their condition is stable, which is beneficial to women's mental state and children's development. In addition, when prescribing drugs for pregnant women with depression, it is important to consider the balance between the benefits and risks of antidepressant use, and it is recommended to avoid prescribing drugs that may have teratogenic effects in clinical practice. Finally, more studies are needed to further elucidate the association between pre-pregnancy/early pregnancy antidepressant use and CHD in children, given the differences in antidepressant classes and CHD phenotypes. It is more critical to study the biological mechanism of antidepressants that may lead to CHD and determine the teratogenic pathway in order to avoid the clinical use of teratogenic drugs.

## Data Availability Statement

The raw data supporting the conclusions of this article will be made available by the authors, without undue reservation.

## Ethics Statement

This study was approved by the Ethics Committee of Xiangya School of Public Health, Central South University, and registered in the Chinese Clinical Trial Registry (Registration Number: ChiCTR1800016635). Prior written informed consent was obtained from all study participants additionally. The patients/participants provided their written informed consent to participate in this study.

## Author Contributions

MS conceptualized the study and drafted the initial manuscript. MS, SZ, and LC analyzed and explained the data. JD, SZ, JL, YLi, LC, YLiu, JW, XS, and JS have collected the data. TW, PH, and JQ conceptualized and designed the study and critically reviewed and revised the manuscript. All authors contributed to the article and approved the final version of the manuscript.

## Funding

This work was supported by National Natural Science Foundation Program of China (82073653 and 81803313), China Postdoctoral Science Foundation (2020M682644), Hunan Provincial Science and Technology Talent Support Project (2020TJ-N07), Hunan Provincial Key Research and Development Program (2018SK2063), Open Project from NHC Key Laboratory of Birth Defect for Research and Prevention (KF2020006), Natural Science Foundation of Hunan Province (2018JJ2551 and 2022JJ40207), Science and Technology Planning Project of Guangdong Province (2020A1414010152), and Changsha Municipal Natural Science Foundation (kq2202470).

## Conflict of Interest

The authors declare that the research was conducted in the absence of any commercial or financial relationships that could be construed as a potential conflict of interest.

## Publisher's Note

All claims expressed in this article are solely those of the authors and do not necessarily represent those of their affiliated organizations, or those of the publisher, the editors and the reviewers. Any product that may be evaluated in this article, or claim that may be made by its manufacturer, is not guaranteed or endorsed by the publisher.
